# Interpretation Criteria for Comparative Intradermal Tuberculin Test for Diagnosis of Bovine Tuberculosis in Cattle in Maroua Area of Cameroon

**DOI:** 10.1155/2016/4834851

**Published:** 2016-08-01

**Authors:** J. Awah-Ndukum, J. Temwa, V. Ngu Ngwa, M. M. Mouiche, D. Iyawa, P. A. Zoli

**Affiliations:** ^1^School of Veterinary Medicine and Sciences, University of Ngaoundéré, BP 454, Ngaoundéré, Cameroon; ^2^Regional Delegation of Livestock, Fisheries, Animal Industries, Far North Region, Cameroon

## Abstract

Intradermal tuberculin test (TST) is the choice method for diagnosis of bovine tuberculosis (Tb) in live animals. This work was done to assess the performance of single intradermal comparative cervical tuberculin (SICCT) test in randomly selected cattle in Maroua, Cameroon, against detection of Tb lesions and detection of Tb lesions plus acid fast bacilli in lesions. While 22.28% of slaughtered cattle presented Tb lesions at meat inspection, detection rates of anti-bovine-Tb antibody, Tb lesions, and Tb lesions plus acid fast bacilli were 68.57%, 32.95%, and 22.35%, respectively. SICCT-bovine-Tb positive cattle were 35.29%, 29.41%, 25.88%, 24.7%, and 21.18% at ≥2 mm, ≥2.5 mm, ≥3 mm, ≥3.5 mm, and ≥4 mm cut-offs, respectively. Higher sensitivity and predictive values were obtained at severe interpretations. The best performance was at ≥3 mm and ≥3.5 mm cut-offs. Against detection of Tb lesions, ≥3 mm and ≥3.5 mm showed sensitivity of 67.8% and specificity of 94.7% and 96.5%, respectively. For detection of Tb lesions accompanied with acid fast bacilli in lesions, ≥3 mm and ≥3.5 mm showed sensitivity of 89.4% and specificity of 92.4% and 93.9%, respectively. These findings revealed that interpretations of SICCT-bovine-Tb should be at ≥3 mm and/or ≥3.5 mm cut-offs. Severe interpretation of TST is essential for optimal diagnosis of bovine Tb in cattle in Maroua, Cameroon.

## 1. Introduction

Bovine tuberculosis (Tb) is a major chronic bacterial disease of animals and humans caused by* Mycobacterium bovis*. Though zoonotic, bovine Tb is neglected and underinvestigated in Sub-Saharan Africa including Cameroon [1, 2]. In areas where bovine Tb is endemic and not controlled or partially controlled, human Tb due to* M. bovis* may occur resulting from ingesting contaminated fresh milk and meat products and by inhaling cough spray from infected cattle [3–8]. Widespread bovine Tb in cattle has been diagnosed in some parts of Cameroon following comparative cervical tuberculin test, detection of Tb lesions during abattoir slaughter meat inspection, acid fast staining of bacilli, and molecular analysis of cultured isolates [1, 9, 10]. Also,* M. bovis* in human has been reported in the West and Northwest Regions of Cameroon [11, 12].

Bovine Tb has significant impact on international trade of livestock and animal products [13]. Intradermal tuberculin skin test (TST) is the international choice method for field diagnosis of bovine Tb in live animals and the World Organisation for Animal Health (OIE) recommended difference between the increases in skin thickness for the test to be positive should be at least 4 mm after 72 hours [13]. However, the performance of TST is affected by environmental and host factors and the nature of the tuberculin used [14–19]. A perfect cut-off point in a specific geographic area or country may not be useful in another environment or another country [14, 17, 20] and the ability of the test to accurately predict true positive disease status depends on its sensitivity, specificity, and prevalence of the disease in the population tested [14]. The OIE recommended cut-off value was established mainly in developed countries for* Bos taurus* cattle and different cut-off values are applied according to a particular country's disease status and objective of its disease control programme [17]. Severe interpretations have been used in Chad, Ethiopia, and Tanzania [15, 17, 21–23] and in regions or herds where* M. bovis* infection had been confirmed based on the discretion of the veterinarian [17].

TST together with slaughter of positive reactors to examine for Tb lesions; culture of suspected Tb specimens; and other modern diagnostic techniques (e.g., gamma-interferon, ESAT-6 tests, and serologic and fluorescence polarization assays) have been compared and are being validated for maximum diagnosis of bovine Tb in cattle in various environmental conditions [14, 24–28]. This study was therefore carried out to estimate the prevalence of bovine Tb and assess the diagnostic performance of TST in the diagnosis of bovine Tb zebu cattle in Maroua area of Cameroon.

## 2. Materials and Methods

### 2.1. Study Area and Population

Cattle from the livestock markets in the environs of Maroua destined for slaughter at the Makabaye abattoir were sampled for the study. About twenty cattle are slaughtered daily in the Makabaye abattoir which provides beef to inhabitants of Maroua city and neighbouring areas (10°30′–10°40′N and 14°20′–14°30′E). A TST bovine Tb prevalence rate of 4.67% (3.89%–5.44%) recorded by Awah-Ndukum et al. [29] in the highlands of Cameroon using OIE recommended standards was used to estimate the number of cattle required to detect at least one positive reactor with 95% confidence and a desired precision of ≥5% as previously described [30]. The selection of cattle for the study was based on haphazard arrival of animals at the abattoir and on random-number generation method of cattle owners from the daily abattoir records whose animals were judged as fit to be slaughtered. However, cattle used for the TST performance study were animals that were judged as fit to be slaughtered and were not slaughtered until at least 72-hour stay at the abattoir.

### 2.2. Detection of Bovine Tuberculosis

During November 2013 to March 2014, blood was collected by venopuncture of the jugular vein from 175 randomly selected cattle intended for slaughter to extract serum for lateral flow assay of anti-BTb antibody (Anti-Bovine Ab®). Single intradermal comparative cervical tuberculin (SICCT) skin test was done on 86 random cattle of the 175 selected animals [13] that were slaughtered at least 72 hours later. Following slaughter of these 175 animals, intensive meat inspections were carried out by JT assisted by veterinary staff of the abattoir based on the government's legislation regulating veterinary health inspection and notification of contagious animal diseases [31]. Evidence of pathologies was also supported by postmortem examination of carcasses as earlier described [32–34]. Briefly, the inspection procedure employed visual examination and palpation of the lungs, liver, and kidneys, lymph nodes of the thoracic and head regions, the mesenteric lymph nodes, and other lymph nodes of the body and various other parts/organs of the carcass.

The sera were extracted and stored at −20°C until analysis was carried out. Similarly, 68 tissues specimens, with suspicious TB lesions (53 thoracic and 7 abdominal lymph nodes and 8 liver tissues) from the 86 SICCT bovine Tb cattle of the 175 slaughtered zebu cattle in the study were collected into sterile plastic containers and also stored at −20°C for up to two months before analysis. Individual animal information such as age estimated by examining the incisors [35], sex, breed [31, 36], and body condition scores [37] was recorded during blood collection. Grinding of TB lesions [38] and direct smear microscopy with Ziehl-Neelsen (ZN) staining for confirmation of acid fast tubercle bacilli and lateral-flow-based rapid test for detection of antibodies in serum were done following standard procedures [13, 39–41] and as described by manufacturer (Anigen Bovine Tb Ab®, BioNote Inc., Korea). Briefly, in the ready-to-use disposable lateral flow kit, 10 *μ*L of test serum was poured into the sample well and, after 1 minute, 3 drops of developing buffer (provided as part of the kit) were placed in the buffer well. The result was interpreted after 20 minutes. The presence of two purple coloured bands within the result window, the test area and control line, indicated antibodies positive result whereas no band in the test area in addition to a visible control purple line was negative. An invalid test was one where no coloured band was visible within the result window. The appearance of a control colour band, for positive or negative assays, indicated that the test was working properly.

Risk assessments of the project were performed by the researchers to avoid hazards to all persons and animals involved in the project. Ethical clearances were obtained from the required authorities before carrying out the study. The purpose of the study was explained to the targeted participants usually with the assistance of resident veterinarians, local leaders at the abattoir, and or trusted intermediaries. An animal was tested after an informed consent was given by the owner. Apart from minor jugular vein puncture for blood collection, intradermal injections of avian and bovine tuberculin, and procedural restraining manipulations for safety purposes, the animals were not subjected to suffering. Slaughtering and dressing of cattle carcasses were done as described by the Cameroon veterinary services [31]. All laboratory analyses including ZN staining were carried out in a laboratory equipped with a category II Biosafety cabinet.

### 2.3. Data Analysis

The data were entered into Microsoft Excel and then transferred to SPSS 20 and R software. Frequency distributions of bovine Tb were generated for the different diagnostic techniques. The Chi-square test was used to evaluate the sensitivity of TST and assess various associations. The ROC (*Receiving Operating Characteristic*) analysis was also used to evaluate diagnostic performance of TST at different cut-off points [30].

## 3. Results 

### 3.1. Prevalence of Bovine Tuberculosis

Over 22.28% (164) of 736 cattle slaughtered at Makabaye-Maroua during the study period presented macroscopic Tb lesions at meat inspection. The cattle with Tb lesions were distributed as follows: 12 of 123 (9.76%) male animals, 152 of 613 (24.80%) female animals, 44 of 133 (33.08%) animals aged 5 to 10 years, 120 of 597 (20.10%) animals aged over 10 years, 62 of 302 (20.53%) Peulh of Sahel zebu, 86 of 242 (35.54%) Bororo/Fulani zebu, and 16 of 192 (8.33%) Toupouri-Massa zebu.

However, 68.57% (95% CI: 61.69–75.45) of 175 randomly selected cattle were positive for anti-bovine Tb antibodies with lateral flow assay. Single intradermal comparative cervical tuberculin (SICCT) skin test done on 86 of these 175 cattle showed 30 (35.29%, 95% CI: 25.1–45.4), 25 (29.41%, 95% CI: 19.7–39.1), 22 (25.88%, 95% CI: 16.6–35.2), 21 (24.7%, 95% CI: 15.5–33.8), and 18 (21.18%, 95% CI: 12.5–29.8) positive reactors at ≥2 mm, ≥2.5 mm, ≥3 mm, ≥3.5 mm, and ≥4 mm cut-off points, respectively. Of the 86 animals, Tb lesions and Tb lesions plus acid fast bacilli were detected in 28 animals (32.95%, 95% CI: 22.95–42.93) and 19 animals (22.35%, 95% CI: 13.5–31.2), respectively. Over 73.26% (63) of the 86 SICCT animals were positive for anti-bovine Tb antibodies corresponding to apparent rates of 3.49%, 31.40%, 29.07%, 25.58%, 24.42%, and 20.93% animals positive for SICCT bovine Tb and anti-bovine Tb antibody at <2 mm, ≥2 mm, ≥2.5 mm, ≥3 mm, ≥3.5 mm, and ≥4 mm cut-off points, respectively.

### 3.2. Diagnostic Performance of Tuberculin Skin Test to Detect Bovine Tuberculosis in Cattle

The performances of SICCT technique at various cut-off points to diagnose bovine Tb in cattle in Maroua, Cameroon, using detection of Tb lesions and detection of Tb lesions accompanied with acid fast bacilli in the lesions as references for defining the status disease are shown in Table 1. Based on computed sensitivity and specificity values of SICCT compared to detection of Tb lesions and Tb lesions plus acid fast bacilli, severe interpretations of SICCT tests detected more diseases cases. Though highest detection of disease cases by SICCT tests was detected at ≥2.0 mm cut-off point, the overall performances were superior at ≥3 mm and ≥3.5 mm cut-off values. The sensitivity of SICCT at ≥3 mm and ≥3.5 mm cut-off points compared to the sensitivity at ≥4 mm cut-off was not significantly higher [*P* > 0.05] against detection of Tb lesions but significantly higher [*P* < 0.05] against detection of Tb lesions plus acid fast bacilli to define disease status.

It is worth mentioning that overall the predictive values were usually superior at SICCT ≥3 mm and ≥3.5 mm cut-off points compared to the OIE recommended (≥4 mm) cut-off point. Indeed, the performance of SICCT against detection of Tb lesions revealed positive predictive values of 73.3 (63.9–82.7); 80.0 (71.5–88.5); 86.3 (78.9–93.6); 90.4 (84.1–96.6); 88.8 (82.1–95.5) and negative predictive values of 89.0 (82.3–95.6); 86.6 (79.3–93.8); 85.7 (78.2–93.1); 85.9 (78.5–93.2); 82.0 (73.8–90.1) at reactors at ≥2 mm, ≥2.5 mm, ≥3 mm, ≥3.5 mm, and ≥4 mm cut-off points, respectively. Accordingly, the performance of SICCT against detection of Tb lesions plus acid fast bacilli revealed positive predictive values of 63.3 (53.05–73.54); 72 (62.45–81.54); 77.3 (68.39–86.20); 81 (72.66–89.34); 77.8 (68.96–86.63) and negative predictive values of 100; 98.3 (95.5–100); 96.8 (93–100); 96.9 (93–100); 92.5 (86.9–98.1).

**Figure 1:**
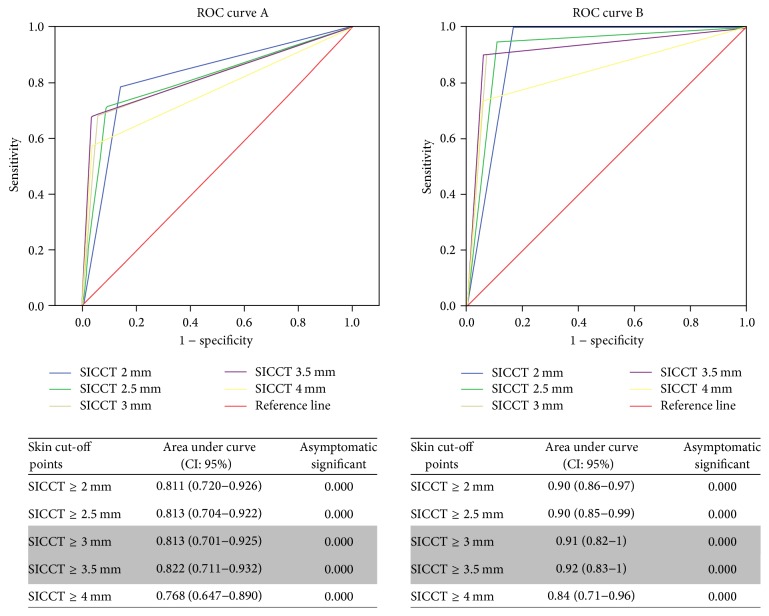
ROC (*Receiving Operating Characteristic*) analysis of the performances of SICCT to detect bovine Tb. Classification of the single intradermal comparative cervical tuberculin (SICCT) skin test cut-off point performance with detection of tuberculous lesions as reference test (curve A). Classification of the single intradermal comparative cervical tuberculin (SICCT) skin test cut-off point performance with detection of tuberculous lesions and acid fast bacilli as reference test (curve B).

Furthermore, the ROC (*Receiving Operating Characteristic*) analysis showed that the area under the curve was significantly higher at cut-off points <4 mm, particularly at ≥3.5 mm cut-off point according to detection of Tb lesions [0.822 (0.711–0.932)] and detection of Tb lesions plus acid fast bacilli in the lesions [0.92 (0.83–1)] [Figure 1]. The area under the ROC curves according to detection of Tb lesions for all SICCT cut-off points was between 0.7 and 0.9 suggesting that these cut-off values are only fairly informative for the detection of bovine Tb. However, SICCT at ≥3.5 mm cut-off point showed significantly higher (*P* < 0.001) discriminatory power compared to SICCT at ≥4 mm cut-off point. For the ROC curves according to detection of detection of Tb lesions plus acid fast bacilli in the lesions, all SICCT cut-off points <4 mm were between 0.9 and 1, particularly for ≥3 mm and ≥3.5 mm cut-off points, indicating that these cut-off values are very informative for the detection of bovine Tb. Therefore, the ROC findings also confirmed severe interpretations of SICCT bovine Tb detection [particularly at ≥3 mm and ≥3.5 mm cut-off points] as for sensitivity and specificity evaluations.

## 4. Discussion

The detection rates of macroscopic Tb lesions [22.28–32.95%] in cattle in this study are much higher than values, ranging from <1 to 4.25%, reported in the littoral and western highland regions of Cameroon [29, 42], while the prevalence of anti-bovine Tb antibodies [68.57% and 73.26%] was higher than 60% recorded in the Bamenda area [42] and 37.17% recorded in the highland regions [43]. Also, significantly higher SICCT bovine Tb prevalence estimates based on tuberculin skin tests at cut-off points ≥4 mm, ≥3 mm, and ≥2 mm were obtained compared to 3.59%–7.48%, 8.92%–13.25%, and 11.77%–17.26% recorded by Awah-Ndukum et al. [43] in the highland regions. However, the rates of SICCT bovine Tb/anti-bovine Tb antibodies animal responses in this study agree with that of Awah-Ndukum et al. [43] who reported that the proportion of SICCT bovine Tb/anti-bovine Tb antibody reactors was significantly higher at the ≥2 mm followed by the ≥3 mm and ≥4 mm cut-off point groups. These findings suggest that bovine Tb is highly endemic in cattle in the Maroua area compared to other parts of Cameroon and require severe interpretations of SICCT bovine Tb results.

Postmortem examination of Tb lesions and demonstration of acid fast bacilli by direct microscopy were used in this study to define disease status of bovine Tb in cattle, to evaluate the performance of tuberculin skin test as opposed to bacteriological culture that was used elsewhere as reference diagnostic test [13]. However, detection of Tb lesions showed lower sensitivity values compared to detection of Tb lesions accompanied with demonstration of acid fast bacilli in the lesions. Macroscopic examination of Tb lesions and demonstration of acid fast bacilli have also been used by Ameni et al. [15] in Ethiopia and Ngandolo et al. [21] in Chad to evaluate the diagnostic performances of tuberculin skin tests. In this study optimal detection of bovine Tb in cattle in Maroua, Cameroon, was obtained at severe interpretations of SICCT and particularly at ≥3 mm and ≥3.5 mm. These findings are similar to those of Ameni et al. [15] who reported that improved diagnostic performances of tuberculin skin test in zebu cattle in Ethiopia were obtained at severe interpretations of >2 mm cut-off point. In Chad, Ngandolo et al. [21] also stated that optimum diagnostic performance of tuberculin skin test in Arab zebus and Bororo zebus was >2 mm cut-off point. The present results agree with those of Awah-Ndukum et al. [43] who observed that improved diagnosis of bovine Tb by tuberculin skin test was obtained at ≥3 mm cut-off when compared to anti-bovine Tb antibody detection in Goudali, Red Bororo, and White Fulani zebus and their crosses in the highlands [Adamawa and Northwest] of Cameroon.

The tuberculin skin tests are currently the best available and affordable techniques for international field diagnosis of bovine TB in live animals [14, 24]. Also, the tests are based on delayed hypersensitivity reactions [13]. The intradermal comparative cervical tuberculin (ICCT) skin test involving the intradermal injection of bovine tuberculin (BT) and avian tuberculin (AT) at separate sites in the skin of the neck gives more specific results than the simple intradermal tuberculin (SIT) skin test which uses only BT [16, 17]. The World Organisation for Animal Health (OIE) recommended difference between the increases in skin thickness for the test to be positive should be >4 mm after 72 hours [13]. However, the OIE recommended cut-off value was established mainly in developed countries for* Bos taurus* cattle [15], in an epidemiologic context of very low prevalence of bovine Tb [≤0.1%] and the implementation of a strict test and slaughter eradication policy [24]. Indeed, different cut-off values have been applied worldwide according to a particular country's disease status and objective of its disease control programme [17]. In Africa, for example, the >2 mm, ≥3 mm, >4 mm, and ≥4 mm cut-off points have been used in Chad, Ethiopia, and Tanzania [15, 17, 22, 23, 44].

The ROC analysis and sensitivity evaluations support severe interpretation of tuberculin skin tests in this study, particularly at ≥3 mm and ≥3.5 mm cut-off points and [43] had proposed severe interpretations of tuberculin skin tests for the diagnosis of bovine Tb in* Bos indicus* cattle in Cameroon, where the prevalence of bovine Tb is high and widespread. The performance of tuberculin skin tests has also been affected by environmental factors, host factors (status of immunity, genetics, etc.), prevalence of the disease in the population tested, and the nature of the tuberculin used [14–19]. A perfect cut-off point in a specific geographic area may not be so useful at another environment [14, 17] and the ability of the test to accurately predict the true positive disease status depends on its sensitivity, specificity, and prevalence of the disease in the population tested [14]. Excessively high sensitivity of tuberculin skin tests will generate false positive reactions during interpretations of test results. However, severe interpretations for improved diagnosis have been done in regions or herds where* M. bovis* infection had been confirmed based on the discretion of the veterinarian [17].

In this study, the best individual sensitivity [67.8% (57.8–77.7) at ≥3.5 mm cut-off point] of tuberculin skin test, with detection of Tb lesions as the reference test, recorded is lower than the median individual sensitivity [80% (52.0–100)] stated by OIE [13] at the recommended >4 mm cut-off point [14]. The best individual sensitivity [89.4% (82.8–95.9) at ≥3.5 mm cut-off point] of tuberculin skin test, with detection of Tb lesions plus acid fast bacilli in lesions as the reference test, recorded is higher than the median individual sensitivity stated by OIE at the recommended cut-off point. The OIE proposed value is a median from a very wide dispersion (52.0–100%) compared to very narrower dispersions for best overall values in the present study (57.8–77.7% and 82.8–95.9%). For SICCT bovine Tb detection, the study showed higher (nonsignificant for detection of Tb lesions and significant for detection of Tb lesions accompanied with AFB in lesions as gold standards) sensitivities at severe (<4 mm cut-off) interpretation compared to interpretation at the OIE recommended (≥4 mm) cut-off value. Severe interpretation of SICCT results diagnosed more bovine Tb cases and is very essential in managing high zoonotic potential [1] as well as high socioeconomic and cultural implication [45] of bovine Tb in Cameroon. The sensitivities obtained in this study are similar to the values of Ameni et al. [15] who reported 68.8% at >2 mm cut-off point in Ethiopia and Delafosse et al. [23] who reported 94% at ≥4 mm in Chad. Various factors can influence the sensitivity of tuberculin skin test and the hypersensitivity reactions can fluctuate considerably depending on the animal. Delayed hypersensitivity reactions provoked by tuberculin injection can become established 3 to 6 weeks after exposure of the host to bacilli agents while recently infected animals may not react sufficiently to tuberculin injection [46]. The reaction is reduced in young animals [calves] and pregnant females [cow] near term [47].

Anergy has been reported to cause false negative reactions during tuberculin skin test but the reasons are still poorly understood [48]. However, recently infected cattle, cattle under stress due to malnutrition, gastrointestinal parasitoses, other concurrent infections, and cattle with generalized Tb would be anergic and fail to react to tuberculin skin test [47, 48]. Therefore, cattle presenting differential SICCT skin thickness of ≤4 mm should not be excluded that they are not affected by bovine Tb, especially animals in highly endemic areas and animals sensitized to environmental mycobacteria such as in Cameroon [29]. These animals could actually be infected but low reacting or not reacting at all because their immune systems may not be sufficiently stimulated for a positive response to occur at the ≥4 mm OIE recommended cut-off point [47, 48]. Also, conditions such as stress may compromise their immune function [49] and animals may be sensitized to environmental mycobacteria [50]. Furthermore, in late stages or towards the end of the course of the disease, the capacities of the infected hosts may become saturated and the expected hypersensitivity reactions may not be observed [51]. Also, 1–5% of some animals may be totally anergic during their entire lifespan [24, 52]. These phenomena are responsible for the fluctuating sensitivities of tuberculin skin tests according to environments and amongst animal populations.

This study revealed that severe interpretation of tuberculin skin tests, at cut-off values less than the OIE recommended cut-off value of >4 mm, is essential for optimal diagnosis of bovine Tb in* Bos indicus* cattle in Maroua, Cameroon. The interpretations should be done at either ≥3 mm or ≥3.5 mm cut-off points given the epidemiological and environmental context of the region.

## Figures and Tables

**Table 1:** Performances of single intradermal comparative cervical tuberculin (SICCT) skin test at various cut-off points to diagnose bovine tuberculosis in zebu cattle in Maroua, Cameroon, using detection of tuberculosis lesions and detection of tuberculosis lesions accompanied with acid fast bacilli in lesions to define disease status.

SICCT cut-off point	Detection of tuberculosis lesions to define disease status				Detection of tuberculosis lesions and acid fast bacilli in lesions to define disease status			
	Sensitivity		Specificity		Sensitivity		Specificity	
	Value, % (95% CI)	*P* value [*χ* ^2^]	Value, % (95% CI)	*P* value [*χ* ^2^]	Value, % (95% CI)	*P* value [*χ* ^2^]	Value, % (95% CI)	*P* value [*χ* ^2^]
≥2 mm	78.5 (69.7–87.2)	0.001^*∗*^ [10.49]	85.9 (78.5–93.2)	0.008^*∗*^ [7.0]	100	0.000^*∗*^ [30.41]	83.3 (75.37–91.23)	0.018^*∗*^ [5.56]
≥2.5 mm	71.4 (61.8–81.0)	0.034^*∗*^ [4.45]	91.2 (85.1–97.2)	0.118 [2.43]	94.7 (89.9–99.4)	0.000^*∗*^ [16.69]	89.4 (82.85–95.94)	0.250 [1.32]
*≥3.0 mm*	*67.8* *(57.8–77.7)*	*0.118 [2.44]*	*94.7* *(89.9–99.4)*	*0.534 [0.38]*	*89.4* *(82.8–95.9)*	*0.004* ^*∗*^ * [8.28]*	*92.4* *(86.76–98.03)*	*0.674 [0.17]*
*≥3.5 mm*	*67.8* *(57.8–77.7)*	*0.118 [2.44]*	*96.5* *(92.6–100)*	*1 [*0*]*	*89.4* *(82.8–95.9)*	*0.004* ^*∗*^ * [8.28]*	*93.9* *(88.81–98.98)*	*1 [*0*]*
≥4 mm	57.1 (46.5–67.6)	/	96.5 (92.6–100)	/	73.6 (64.2–82.9)	/	93.9 (88.81–98.98)	/

^*∗*^Significantly different [*P* < 0.05] when compared to ≥4 mm cut-off point.
